# Limited Diversity of Thermal Adaptation to a Critical Temperature in *Zymomonas mobilis*: Evidence from Multiple-Parallel Laboratory Evolution Experiments

**DOI:** 10.3390/ijms26073052

**Published:** 2025-03-26

**Authors:** Sornsiri Pattanakittivorakul, Shun Kato, Takashi Kuga, Tomoyuki Kosaka, Minenosuke Matsutani, Masayuki Murata, Morio Ishikawa, Kankanok Charoenpunthuwong, Pornthap Thanonkeo, Mamoru Yamada

**Affiliations:** 1 Graduate School of Sciences and Technology for Innovation, Yamaguchi University, Yamaguchi 753-8515, Japan; sornkiri@gmail.com (S.P.); sh.kato@ikedatohka.co.jp (S.K.); t.kuga0724@gmail.com (T.K.); tkosaka@yamaguchi-u.ac.jp (T.K.); murama0054@gmail.com (M.M.); 2 Research Center for Thermotolerant Microbial Resources, Yamaguchi University, Yamaguchi 753-8515, Japan; 3 NODAI Genome Research Center, Tokyo University of Agriculture, Tokyo 156-8502, Japan; mine@yamaguchi-u.ac.jp; 4 Department of Bioscience, Tokyo University of Agriculture, Tokyo 156-8502, Japan; m1ishika@nodai.ac.jp; 5 Department of Biotechnology, Faculty of Technology, Khon Kaen University, Khon Kaen 40002, Thailand; cchomkan@gmail.com (K.C.); portha@kku.ac.th (P.T.); 6 Fermentation Research Center for Value Added Agricultural Products, Khon Kaen University, Khon Kaen 40002, Thailand

**Keywords:** laboratory evolution, *Zymomonas mobilis*, limited diversity of thermal adaptation, transcriptomic analysis

## Abstract

Laboratory evolution is an effective means of understanding microbial adaptation to the environment. We previously isolated four thermoadapted *Zymomonas mobilis* mutants, which showed a 2 °C rise in the critical high temperature (CHT), by performing multiple-parallel adaptation experiments. In the present study, the individual mutations in these mutants were intensively analyzed. Two mutations in each adapted mutant were found to primarily contribute to the increase in the upper temperature limit. RNA sequencing (RNA-seq) analysis revealed that the two mutations led to the upregulation of 79–185 genes and the downregulation of 242–311 genes. The findings from transcriptomic and physiological experiments suggest two common and primary mechanisms for thermal resistance: a decrease in the activity of diacylglycerol kinase, which may change the structure of lipopolysaccharide (LPS) probably to strengthen the membrane structure, and an increase in the expression of genes for GroEL/GroES or cell wall hydrolase to repair the protein or membrane damage that occurs at such critical temperatures. Additionally, transporters including efflux pumps may contribute to intracellular homeostasis by expelling toxic compounds such as ethanol and acetate or by maintaining the K^+^ concentration. The results of this study on four independently thermoadapted mutants led to the conclusion that the mutants have almost the same thermal adaptation strategies and thus their molecular diversity is limited.

## 1. Introduction

Microbial adaptation to high temperatures represents a fundamental aspect of evolutionary biology with significant implications for understanding stress response mechanisms. Mesophilic microorganisms that have adapted to elevated temperatures in tropical environments or in the intestines of homeothermic animals serve as valuable models for studying these adaptive processes. Genes responsible for thermotolerance in microorganisms—including *Escherichia coli* and thermotolerant *Acetobacter tropicalis* and *Zymomonas mobilis* isolated in Thailand—have been comprehensively analyzed [1,2,3]. These thermotolerant genes fall into nine functional categories: general metabolism, membrane stabilization, transporters, DNA repair, tRNA/rRNA modification, protein quality control, translation control, cell division, transcriptional regulation, and others. Notably, seven conserved thermotolerant genes are shared among these microorganisms [2]. These findings suggest common thermotolerance mechanisms despite their different CHTs as upper limits of survival [3]. These mechanisms primarily contribute to membrane stabilization, protection, and the repair of macromolecular damage and the maintenance of cellular metabolism at CHTs [2].

Laboratory evolution experiments have emerged as powerful tools for investigating microbial adaptation to thermal stress under controlled conditions [4,5,6]. These experiments typically follow two main approaches: repetitive cultivation at fixed temperatures or stepwise temperature increases to identify adaptive capabilities and limitations. The laboratory evolution of *E. coli* has been attempted in various studies under different thermal conditions. Experiments have been conducted through repetitive cultivations in minimum or rich media at fixed temperatures ranging from 20 °C to 42 °C [7,8,9,10] or through stepwise temperature increases reaching up to 44.8 °C [11] or 48.5 °C [12,13]. Studies focusing on fixed-temperature adaptation have aimed to elucidate microbial adaptation to specific environments [7], estimate the number and diversity of beneficial mutations [8], determine the proportion of beneficial mutations in evolving lineages [9], and examine processes governing evolutionary convergence and divergence [10]. Thermoadapted mutants isolated through stepwise temperature increases in rich media have demonstrated survival at equivalent high temperatures in minimum media [12], suggesting fundamental rather than nutrient-dependent adaptations. Recent work with multiple microbial species, including two *Z. mobilis* strains and one *E. coli* strain, has revealed that thermoadapted mutants could increase their CHTs by approximately 2 °C, with an additional 1 °C improvement achieved in a *Z. mobilis* mutant through further adaptation using a mutator strain [14]. These findings suggest the existence of adaptation limits that likely reflect fundamental biophysical constraints.

Despite significant advances, important knowledge gaps remain in our understanding of thermal adaptation. Although genetic differences among strains evolved from multiple parallel cultivation lines have been analyzed [7,12], detailed information at the individual mutation level—which is crucial for understanding the diversity of thermal adaptations—remains limited. Many evolution studies have been conducted at temperatures significantly below the CHT of ancestral strains [7,8,9,10], potentially failing to capture the full spectrum of adaptations required at thermal extremes. Furthermore, the extent to which laboratory evolution experiments recapitulate natural thermal adaptation processes remains poorly understood.

This study represents, to the best of our knowledge, the first systematic investigation into the diversity of thermal adaptation mechanisms at the CHT in *Z. mobilis* through laboratory evolution. We employed a multi-faceted approach involving the independent isolation of four thermoadapted mutants from four parallel adaptation lines, the transfer of each mutation into the genome of the ancestral strain, and a detailed assessment of mutation effects through transcriptomic analysis and physiological comparisons. This comprehensive approach allowed us to evaluate whether thermal adaptation at the molecular level exhibits high diversity (multiple distinct solutions to thermal stress) or limited diversity (convergent evolution toward similar solutions). Our findings suggest that the four thermoadapted mutants employ remarkably similar molecular mechanisms for thermal adaptation, indicating the relatively limited diversity of adaptive strategies.

Importantly, we observed substantial overlap between laboratory-evolved thermotolerance mechanisms and those occurring naturally in the wild, suggesting that laboratory evolution effectively recapitulates key aspects of natural adaptation to high temperatures. Through elucidating these fundamental principles of thermal adaptation in *Z. mobilis*, our study provides critical insights into the constraints and mechanisms of microbial evolution under thermal stress—with potential implications for understanding adaptation processes across diverse microbial systems. The practical significance of this research extends beyond theoretical understanding, as enhanced thermal adaptation in fermentative microorganisms such as *Z. mobilis* increases their thermostability, making them more resistant to fermentation heat—a crucial advantage for industrial applications [15,16,17,18]. Fermentation at higher-than-conventional temperatures offers several economic and operational benefits, including reduced cooling costs, decreased requirements for saccharifying enzymes, and the suppression of bacterial contamination. Furthermore, the heat-resistant strains developed in this study demonstrate increased tolerance to multiple stresses beyond temperature, providing additional advantages for fermentation production; therefore, both the thermotolerant strains and the knowledge gained from this research represent valuable resources for improving ethanol production using *Z. mobilis*.

## 2. Results

### 2.1. Identification of Mutations That Caused an Increase in Thermal Resistance

Through inducing thermal adaptation with a stepwise temperature increase in four lines of *Z. mobilis* CP4 in parallel, we obtained one thermoadapted mutant from each line, yielding four mutants (Z4-80a, Z4-80b, Z4-80c, Z4-80d) in total. The four mutants were subjected to next-generation sequencing to find mutation points [14]. The consequent amino acid substitutions and insertions are shown in Table 1. Interestingly, Z4-80a, Z4-80c, and Z4-80d have different missense mutations in ZCP4_RS08695 (*dgkA*) for diacylglycerol kinase. Z4-80a and Z4-80c have the same missense mutation in ZCP4_RS02830 (*rpoB*) for RNA polymerase subunit beta, different missense mutations in ZCP4_RS00655 for the potassium transporter Kup, and different missense mutations in ZCP4_RS08505 for an RND family efflux transporter.

**Figure 1:**
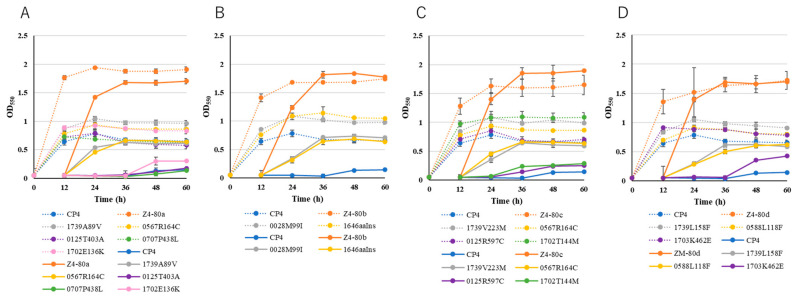
A two-step cultivation assay to examine the effects of individual mutations in thermoadapted mutants on growth at 38 °C. Single-mutation mutants generated from Z4-80a (**A**), Z4-80b (**B**), Z4-80c (**C**), and Z4-80d (**D**) were grown in the first cultivation (dashed lines) and in the second cultivation (straight lines) with each thermoadapted mutant and the parental strain as a control under the conditions described in Materials and Methods.

In order to identify the mutations that confer thermal resistance to survive at temperatures over the CHT of the parental strain, we constructed single-gene mutants by introducing individual mutations in the protein-coding region of the four thermoadapted mutants into the genome of the parental strain (see the table in Section 4.1). The generated single-mutation mutants were named by combining the number of old locus tags with amino acid substitutions or insertions (aaIns). The effect of each mutation on growth at the upper temperature limit was examined using the two-step culture method [14] at 38 °C, which is 1 °C higher than the CHT of the parental strain but 1 °C lower than the CHTs of the four thermoadapted mutants (Figure 1).

Five single-mutation mutants, 1739A89V, 0567R164C, 0125T403A, 0707P438L, and 1702E136K, and Z4-80a were cultured to compare their turbidities with that of the parental strain (Figure 1A). In the second cultivation, 1739A89V and 0567R164C showed clearly higher turbidities than the other strains except for Z4-80a, reaching OD_550_ values of 0.63 and 0.66 at 36 h compared to 0.13 for the parental strain. The results suggest that the mutations in 1739A89V and 0567R164C are primarily responsible for the thermal resistance acquired by Z4-80a. Similarly, 0028M99I and 1646aaIns, 1739V223M and 0567R164C, and 1739L158F and 0588L118F showed higher turbidities (Figure 1B–D) with OD_550_ values increased by 14–20 times higher than that of the parental strain at 36 h, and may be primarily responsible for the thermal resistance acquired by Z4-80b, Z4-80c, and Z4-80d, respectively. On the other hand, mutants for the potassium transporter, ABC transporter, or efflux pumps showed turbidities slightly higher than or similar to that of the parental strain. Therefore, it is likely that two mutations mainly contribute to the thermal resistance of each adapted mutant and that mutations in transporters had little effect on thermal resistance.

### 2.2. RNA-Seq Analysis of Thermoadapted Mutants and Single-Mutation Mutants

The discovery that two mutations were involved in acquired thermal resistance in each adapted mutant motivated us to perform a genome-wide gene expression analysis (RNA-seq). The analysis was performed on the four thermoadapted mutants, Z4-80a, Z4-80b, Z4-80c, and Z4-80d, and seven single-mutation mutants, 1739A89V, 0567R164C, 0028M99I, 1646aaIns, 1739V223M, 1739L158F, and 0588L118F, each of which has a mutation conferring thermal resistance, as well as CP4. The number of single-mutation mutants was seven, not eight, because 0567R164C was shared by Z4-80a and Z4-80c (Table 1). RNA was prepared from cells that had been grown in YPD medium for 8 h at 37 *°*C, which is the CHT for CP4 as the parental strain.

Z4-80a, Z4-80b, Z4-80c, and Z4-80d were found to have 309, 95, 310, and 104 upregulated differentially expressed genes (DEGs) and 372, 100, 382, and 189 downregulated DEGs, respectively. Venn diagram analysis revealed that 72 DEGs were conserved in the four thermoadapted mutants, including 28 upregulated and 45 downregulated genes (Supplementary Materials, Figure S1 and Table S1). The genes with increased expression encode eight ribosomal proteins and one protein synthesis initiation factor, implying that stable protein synthesis is crucial for growth at high temperatures. The genes with downregulated expression encode three ATP-dependent transporters and one ATP-dependent protease, suggesting that energy saving occurs at high temperatures. To understand how many common pathways were differentially expressed in the thermoadapted mutants, Gene Ontology (GO) enrichment analysis was carried out and the top 20 enriched pathways are listed in Table S2. In the case of upregulated DEGs, seven pathways are shared by all four thermoadapted mutants: cellular anatomical entity, cellular process, metabolic process, catalytic activity, binding, cytoplasm, and membrane; six pathways are shared by three thermoadapted mutants: cellular metabolic process, intracellular, ion binding, organic cyclic compound binding, heterocyclic compound binding, and cell periphery. In the case of downregulated DEGs, five pathways are shared by four thermoadapted mutants, cellular anatomical entity, cellular process, membrane, cell periphery, and transport; eight pathways are shared by three thermoadapted mutants: metabolic process, catalytic activity, binding, intracellular, cytoplasm, intrinsic component of membrane, plasma membrane, and localization. These high levels of shared pathways in GO enrichment suggest that the four thermoadapted mutants have acquired similar metabolic changes.

RNA-seq analysis of the single-mutation mutants revealed changes in the expression of many genes—including 165 upregulated and 299 downregulated DEGs—in 0567R164C, which has a missense mutation in *rpoB* (Supplementary Materials Figure S2). Similarly, changes in the expression of many genes, including 47 upregulated and 215 downregulated DEGs, were observed in 0028M99I, which has a missense mutation in ZCP4_RS00160 for a sensor histidine kinase of a two-component regulatory system involved in signal transduction [19,20]. Surprisingly, 0588L118F and 1646aaIns affected the expression of many genes, including 84 upregulated and 260 downregulated DEGs and 48 upregulated and 87 downregulated DEGs, respectively; 0588L118F has a missense mutation in ZCP4_RS02940 (*purN*) for phosphoribosyl glycinamide formyltransferase [21], which is involved in purine metabolism, and 1646aaIns has an insertion corresponding to six amino acid residues (IYDGSL insertion) in ZCP4_RS08220 (*hisA*) for 1-(5-phosphoribosyl)-5-[(5-phosphoribosyl amino)methylideneamino] imidazole-4-carboxamide isomerase, which is involved in histidine metabolism [22].

### 2.3. Factors for Acquired Thermal Resistance

As mentioned above, each thermoadapted mutant has two mutations primarily responsible for acquired thermal resistance. Venn diagram analysis was thus performed to explore factors for acquired thermal resistance (Supplementary Materials Figure S2). First, we focused on the finding that three of the four thermoadapted mutants have a mutation in *dgkA*, although the mutation sites are different. However, the number of DEGs was relatively small in the three *dgkA* mutants, 1739A89V, 1739V223M, and 1739L158F, and there were no common DEGs when DEGs shared by Z4-80a and 1739A89V, DEGs shared by Z4-80c and 1739V223M, and DEGs shared by Z4-80d and 1739L158F were compared (Supplementary Materials Table S3).

We carefully examined the RNA-seq data for *dgkA* expression changes across mutant strains (Table 2). Our analysis revealed distinct patterns of *dgkA* regulation. In Z4-80b (which lacks mutations in *dgkA* itself) and in 0028M99I, *dgkA* expression decreased. Conversely, we observed increased *dgkA* expression in Z4-80a, Z4-80c, Z4-80d, 1739A89V, and 1739V223M. These findings suggest two different mechanisms affecting diacylglycerol kinase activity. In Z4-80a, Z4-80c, and Z4-80d, the *dgkA* mutations likely directly alter the enzyme’s activity. In contrast, the reduced diacylglycerol kinase activity in Z4-80b appears to result from decreased gene expression instead. The increased expression observed in several mutants may represent a compensatory response to altered diacylglycerol kinase function, though this upregulation appears insufficient to restore normal activity in strains carrying *dgkA* missense mutations. Additionally, we examined a second diacylglycerol kinase gene in *Z. mobilis* CP4, *ZCP4_RS08515* (*dgkB*). This gene was downregulated in multiple thermoadapted strains: Z4-80a, Z4-80c, Z4-80d, 0028M99I, 0567R164C, and 0588L118F (Table 3). Based on these expression patterns and the known role of diacylglycerol kinase in lipid metabolism, we hypothesize that altered membrane lipid composition—potentially including LPS modifications—may contribute to the enhanced thermal resistance in these mutants.

While direct structural analysis of LPS was beyond the scope of the current study, our physiological and transcriptional data provide a foundation for future investigations using mass spectrometry or other direct structural validation methods to confirm the hypothesized changes in LPS composition resulting from altered diacylglycerol kinase activity.

**Figure 2:**
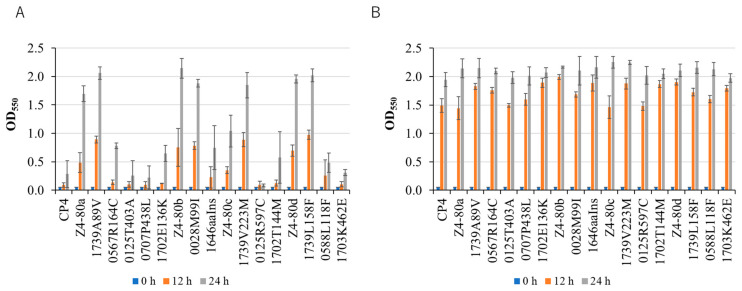
The effects of individual mutations in thermoadapted mutants on growth in the presence of polymyxin B. Single-mutation mutants generated from Z4-80a, Z4-80b, Z4-80c, and Z4-80d were grown at 30 °C in YPD medium supplemented with (**A**) and without (**B**) 1 μg/mL polymyxin B. Cell growth was estimated by measuring turbidity at OD_550_.

The activity of diacylglycerol kinase, which catalyzes the ATP-dependent phosphorylation of diacylglycerol to convert phosphatidic acid, influences the LPS structure in *E. coli* [23,24], and the deletion of *dgkA* in a polymyxin-resistant strain results in a severe reduction in phosphoethanolamine modification in LPS and the loss of the polymyxin B resistance. We thus examined polymyxin B sensitivity in all of the thermoadapted mutants and single-mutation mutants. Cells were grown at 30 °C in YPD medium supplemented with 1 μg/mL polymyxin B or in YPD medium alone as a control, and cell growth was estimated by measuring turbidity (Figure 2). All of the thermoadapted strains and 1739A89V, 1739V223M, 1739L158F, and 0028M99I showed much higher growth, over 1.5 (OD_550_) at 24 h, than that of CP4, less than 0.5 (OD_550_), indicating resistance to polymyxin B, and 0567R164C and 1646aaIns were slightly resistant to the drug. Therefore, the phenotype of resistance to polymyxin B may be due to the reduced activity of diacylglycerol kinase due to mutations in *dgkA* or the reduced expression of *dgkA*. Although it is unclear why *dgkA* mutations or downregulation in *Z. mobilis* led to a polymyxin B resistance phenotype opposite to that caused by the deletion mutation in *dgkA* in the polymyxin-resistant *E. coli* strain, the structural change in LPS may lead to a more rigid outer membrane. Consistent with this, *dgkA* mutations or downregulation gave rise to increased resistance to ethanol or acetic acid, as mentioned below.

We explored another factor for acquired thermal resistance in the remaining mutants, 0567R164C, 1646aaIns, and 0588L118F, each of which has a mutation for thermal resistance, and we found a set of chaperonin GroEL and co-chaperone GroES [25,26] or cell wall hydrolase [27]. RNA-seq analysis revealed the increased expression of *groEL* and *groES* in 0567R164C and 1646aaIns, as well as in Z4-80a, Z4-80b, and Z4-80c (Table 3). Therefore, the missense mutation in *rpoB* in Z4-80a and Z4-80c and the insertion mutation of *hisA* in Z4-80b may cause the increased expression of *groEL* and *groES*. Notably, Rudolph et al. [12] reported that GroEL and GroES appear to be mediators of the evolution of extremely heat-resistant *E. coli* cells, and Sakunda et al. [28] showed that the overexpression of *groEL* and *groES* causes a 1 °C increase in the CHT of *Z. mobilis*. On the other hand, since neither gene was upregulated in Z4-80d, we carefully looked for other candidates in the mutant, which should be present in upregulated DEGs shared by Z4-80d and 0588L118F and absent from upregulated DEGs shared by Z4-80a and 0567R164C, Z4-80b and 0028M99I or 1646aaIns, and Z4-80c and 0567R164C (Supplementary Materials Table S4). Of these, the genes for fundamental metabolism, *ZCP4_RS01475*, *ZCP4_RS03815*, *ZCP4_RS03820*, and *ZCP4_RS05995*, can be excluded because their upregulation should be observed in other thermoadapted mutants if they are needed for growth at the CHT. Genes for transcriptional regulators can also be excluded because their target genes should be present in the list if they are key factors. Therefore, the upregulation of *ZCP4_RS04150* for cell wall hydrolase is assumed to be another factor for acquired thermal resistance in Z4-80d. Cell wall hydrolases may play important roles in survival at the CHT because they are involved in quality control or remodeling of the cell wall under environmental stress [27,29].

### 2.4. Minor but Important Contribution of Transporters to Thermal Adaptation

As mentioned above, two mutations in each adapted mutant shown at the top of Table 1 contributed to the acquired thermal resistance. All remaining mutations occurred in genes for transporters, and thus, transport activity is assumed to be important for thermal adaptation. Z4-80a and Z4-80c have mutations in *ZCP4_RS00655* that result in amino acid substitutions in Kup, which facilitates K^+^ uptake via proton cotransport [30]. Considering evidence showing that the addition of exogenous K^+^ increases the CHT by 1 °C in *Z. mobilis* [31], the mutations in *ZCP4_RS00655* may cause an increase in the uptake of K^+^ by, for example, changing the affinity for the ion. However, the effect of the *ZCP4_RS00655* mutations may not be strong enough to elevate the CHT by 1 °C as in the case of exogenous K^+^.

**Figure 3:**
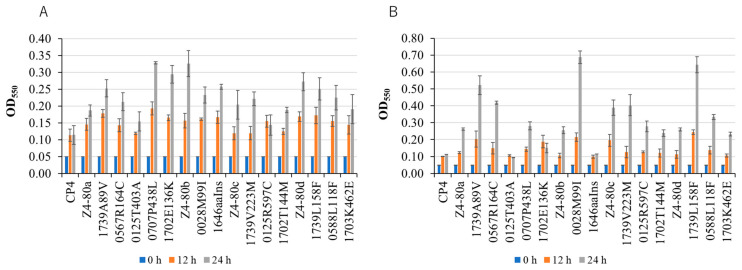
The effects of individual mutations in thermoadapted mutants on growth in the presence of exogenous ethanol or acetic acid. Single-mutation mutants generated from Z4-80a, Z4-80b, Z4-80c, and Z4-80d were grown at 30 °C in YPD medium supplemented with 10% (*w*/*v*) ethanol (**A**) and 5% (*w*/*v*) acetic acid (**B**). Cell growth was estimated by measuring turbidity at OD_550_.

The gene organization of the genome (GenBank) suggests that *ZCP4_RS08505* and *ZCP4_RS08510* with *ZCP4_RS08495* compose an operon, which encodes a tripartite efflux pump [32]. *ZCP4_RS08495*, *ZCP4_RS08505*, and *ZCP4_RS08510* encode for an inner membrane protein (IMP) annotated as a fusaric acid resistance family protein, a periplasmic adapter protein (PAP) annotated as an HlyD family secretion protein, and an outer membrane factor (OMF) annotated as a TolC family protein, respectively, of the efflux pump. Notably, this operon has one more cistron, *ZCP4_RS08500*, encoding a DUF1656 domain-containing protein, which is suggested by its upregulation together with the other three genes (Table 4), and the efflux pump may thus consist of four proteins. Considering that ethanol and acetic acid are compounds that inhibit *Z. mobilis* cells, especially under fermentation conditions, we examined the effects of missense mutations in *ZCP4_RS08505* in Z4-80a and Z4-80c and *ZCP4_RS08510* in Z4-80d on growth at 30 °C in YPD medium supplemented with 10% (*w*/*v*) ethanol or 5% (*w*/*v*) acetic acid. As expected, 1702E136K, 1702T144M, and 1703K463E showed better growth at 12 or 24 h, about two times higher turbidity (OD_550_) than that of the parental strain (Figure 3). Therefore, these may be gain-of-function mutations that enhance efflux activity. *ZCP4_02520*, *ZCP4_02525*, *ZCP4_02530*, and *ZCP4_02535* constitute a set of genes for another efflux pump, which consists of a PAP annotated as an HlyD family protein, an ATPase/putative transporter RbbA, an IMP annotated as an ABC transporter permease, and an OMF annotated as a TolC family protein and were found to be upregulated in all thermoadapted mutants (Table 4). *ZCP4_08380*, a gene for a major facilitator superfamily (MFS) transporter, was upregulated in all thermoadapted mutants, and *ZCP4_RS03940* and *ZCP4_RS08395* for MFS transporters were upregulated in more than two thermoadapted mutants (Table 4). Some MFS transporters may form a complex with the PAP and OMF to function as a tripartite efflux pump [32].

There was a missense mutation in *ZCP4_03540* in Z4-80a (Table 1). The gene organization of the genome suggests that *ZCP4_03540* composes an operon with *ZCP4_03545*. The former and latter encode a nitrate/sulfonate/bicarbonate ABC transporter ATP-binding protein and permease subunit, respectively. The missense mutation led to resistance to ethanol and acetic acid (Figure 3). In the presence of 10% (*w*/*v*) ethanol, the turbidity at OD_550_ of 0707P438L was two and three times higher than that of the parental strain at 12 and 24 h, respectively, and in the presence of 5% (*w*/*v*) acetic acid, the turbidity of 0707P438L was 1.5 and 2.6 times higher than that of the parental strain at 12 and 24 h, respectively. Therefore, these findings allow us to speculate that the transporter functions as an exporter [33] to remove some of the inhibitory compounds from cells. However, one or both of *ZCP4_03540* and *ZCP4_03545* were significantly or slightly downregulated in Z4-80a, Z4-80b, and Z4-80c (Table 2 and Table 4). The downregulation of both genes might contribute to saving ATP. On the other hand, *ZCP4_RS07805* for the ABC transporter permease was upregulated in more than two thermoadapted mutants.

## 3. Discussion

Our previous research showed that the potential increase in CHTs in *Z. mobilis* and *E. coli* is approximately 2 °C and that the application of a mutator can further increase the CHT by 1 °C [14]. Similar increases in the CHT in *E. coli* have been suggested by thermal adaptation experiments with a stepwise increase in temperature [12,13]. In this study, in order to understand the diversity of thermal adaptation, we identified mutations that are responsible for raising the CHT in four thermoadapted mutants, which were isolated by performing parallel thermal adaptation experiments, and we further investigated the impact of each effective mutation via transcriptomic and physiological analyses. Our experimental evidence demonstrates that each thermoadapted mutant has two mutations responsible for the increase in the CHT. Transcriptomic analysis revealed a substantial overlap of DEGs among the four thermoadapted mutants: 9–29% of upregulated genes and 12–45% of downregulated genes. Notably, mutations in 0567R164C, 0028M99I, 1646aaIns, and 0588L118F, which contribute to the thermal resistance of the corresponding thermoadapted mutant, influence many shared genes (33–79% of upregulated genes and 68–79% of downregulated genes between two mutants) (Table 5). GO enrichment analysis further confirmed that all four thermoadapted mutants exhibit DEGs in common pathways (Supplementary Materials Table S2). These findings imply that the four thermoadapted mutants fundamentally have a common molecular strategy for thermal adaptation.

Detailed analysis revealed that all four thermoadapted mutants acquired similar thermal resistance mechanisms during adaptation, primarily through two key factors. The first common factor involves decreased diacylglycerol kinase activity, resulting from either mutations in or downregulation of the *dgkA* gene. This modification leads to structural changes in the outer membrane, conferring resistance to polymyxin B, acetic acid, and ethanol (Figure 2 and Figure 3), thus essentially reinforcing the outer membrane barrier. The second factor manifests as either increased expression of *groEL*/*groES* (observed in three of the thermoadapted mutants) or enhanced expression of the cell wall hydrolase gene *ZCP4_RS04150* (in the remaining mutant). This factor is associated with repairing the protein and cell wall damage that occurs at challenging high temperatures, thus strengthening repair functions.

If the specific role of GroEL/GroES involves quality control of cell wall hydrolase at challenging high temperatures, these seemingly different adaptations may converge into a unified mechanism: the maintenance of effective cell wall repair. This convergence hypothesis is supported by our observation that the Z4-80c mutant, which showed relatively modest upregulation of *groEL*/*groES*, compensated with significantly greater upregulation of the cell wall hydrolase gene (Table 3). The notable similarity in gene expression patterns across thermoadapted mutants and single-mutation mutants provides compelling evidence that thermal resistance mechanisms in *Z. mobilis* demonstrate remarkably limited diversity, suggesting evolutionary constraints on pathways for adapting to thermal stress.

Thermal adaptation has been attempted in four lines of *E. coli*, resulting in two thermoadapted mutants with a 2 °C increase in CHT, while the other two lines died out [14]. Both surviving mutants contained missense mutations in *spoT*, which encodes the bifunctional (p)ppGpp synthetase II and guanosine-3′,5′-bis pyrophosphate 3′-pyrophosphohydrolase. This enzyme controls intracellular ppGpp levels, influencing the expression of numerous genes [34,35]. Similar *spoT* mutations have been identified in other thermoadapted *E. coli* strains [9,13], suggesting limited diversity in *E. coli* thermal adaptation mechanisms. Interestingly, one of these two *E. coli* thermoadapted mutants also contained a missense mutation in *rpoC* (encoding the RNA polymerase beta prime subunit), while the other had a mutation in *trkH* (encoding a K^+^ transporter) [14]. These mutations parallel those found in thermoadapted *Z. mobilis* strains Z4-80a and Z4-80c. Previous research has established that genes encoding RNA polymerase components, including *rpoB* and *rpoC*, are primary targets for thermal adaptation in *E. coli* [7]. Additionally, studies have shown that GroEL/GroES chaperones are essential for folding mutated proteins generated during *E. coli* evolution [12]. These findings align with observations in *Z. mobilis* strains Z4-80a and Z4-80c, and partially in Z4-80b. Similarly, a thermoadapted mutant of *Acetobacter pasteurianus* exhibited missense mutations in *spoT*, *rpoC*, and two genes encoding a two-component hybrid sensor histidine kinase and regulator [36]. Analogous mutations were observed in thermoadapted *E. coli* strains and *Z. mobilis* Z4-80b [14]. Blaby et al. [13] further confirmed *spoT* missense mutations in thermoadapted *E. coli* strains. The occurrence of overlapping mutations across different bacterial species suggests common pathways for thermal adaptation. These mutations likely affect global gene expression patterns, indicating the existence of shared adaptive mechanisms across diverse bacterial taxa.

Taken together, the results of laboratory evolution experiments suggest the capacity of *Z. mobilis* or *E. coli* to have a narrow range of CHT increases and limited diversity, raising the following concerns. As global warming progresses, mesophilic bacteria with a low capacity for thermal adaptation may become extinct when exposed to temperatures over individual adaptation limits. Their extinction would have a negative impact on biogeochemical cycles. In addition, pathogenic bacteria such as *Salmonella typhimurium*, *Pseudomonas aeruginosa*, and *Yersinia pestis*, which may have adapted to high temperatures in their hosts possess a set of LPS sugar modification genes and a set of sulfur relay genes, both of which have been discovered to be thermotolerance genes in *E. coli* [3] but are absent in most of mesophilic, non-pathogenic bacteria. This may disrupt the microbial balance and cause the spread of pathogens.

The outer membrane reinforcement and repair function enhancement observed in this study appear to overlap with previously proposed thermotolerance mechanisms essential for survival at CHTs [3]. These mechanisms have been identified through analyses of three thermotolerant bacterial species [1,2,3]. Matsumoto et al. [37] demonstrated that mutations in three specific genes enhanced thermotolerance in *A. pasteurianus* TH-3 by improving cell surface integrity.

The established thermotolerance mechanism encompasses several components: membrane stabilization, reactive oxygen species (ROS) scavenging, repair of damaged macromolecules (including DNA and proteins), and stabilization of tRNA structure [3]. Specifically, outer membrane reinforcement contributes to inner membrane stabilization, preventing electron leakage from the respiratory chain at elevated temperatures, thereby suppressing ROS generation [3,38]. As ROS damage cellular macromolecules, reducing their production likely promotes cell growth at higher temperatures. This hypothesis is supported by Matsumoto et al. [39], who demonstrated that decreased ROS levels enhanced thermal resistance in an *A. pasteurianus* NBRC 3283 mutant. Complementarily, enhanced repair functions reduce the accumulation of damaged macromolecules at high temperatures. These findings indicate that the thermotolerance mechanisms naturally acquired by microorganisms in high-temperature environments align well with our laboratory thermal adaptation results. We can, therefore, conclude that thermoadapted mutants have evolved to incorporate specific components of the broader thermotolerance mechanism.

In essence, laboratory thermal adaptation appears to replicate aspects of long-term adaptation to high-temperature environments observed in nature. However, it is important to note that laboratory conditions represent a simplified version of natural adaptation, with controlled medium composition and culture conditions and, notably, an absence of microbial interactions. This suggests that more complex laboratory thermal adaptation approaches may be necessary to fully replicate natural evolutionary processes.

In addition to mutations directly related to thermal resistance, mutations in potassium transporters and efflux pumps were found in two or three thermoadapted mutants. As mentioned in the results section, most of these mutations are assumed to be gain-of-function adaptations that maintain intracellular K^+^ concentration at high temperatures [31] or expel various compounds that have a negative impact on cell viability. Therefore, these mutations may contribute to the maintenance of homeostasis at elevated CHTs. Although it is not clear which mutation for outer membrane reinforcement or repair function reinforcement was acquired first, each mutation is thought to elevate the CHT by about 1 °C. It is also unclear when these mutations and transporter mutations were introduced, but some of them might be correlated. In Z4-80b, the mutation in 0028M99I not only decreases the expression of *dgkA*, leading to outer membrane reinforcement (Table 3), but also influences the expression of genes for efflux pumps (Table 4). On the other hand, the *dgkA* mutations in Z4-80a, Z4-80c, and Z4-80d lead to outer membrane reinforcement but hardly influence the expression of genes for efflux pumps, except for *ZCP4_RS08380*. It is, therefore, speculated that the *dgkA* mutations and efflux pump mutations may have been introduced simultaneously. There are mutations in *ZCP4_RS00655* for the potassium transporter only in Z4-80a and Z4-80c, but there is little change in the expression of *ZCP4_RS00655* in all thermoadapted mutants (Table 2). Since RNA polymerase activity requires K^+^ [40], it is possible that RNA polymerases with missense mutations in Z4-80a and Z4-80c increase the K^+^ requirement to gain mutations in the K^+^ transporter, and thus, both mutations might have occurred at nearly the same time.

Although it is understandable that the mutation in His kinase in the two-component regulatory system and the mutations in the RNA polymerase subunit affected the expression of many genes, it is surprising that the 6aa insertion mutation and missense mutation in HisA and PurN, respectively, influenced the expression of many genes (Figure S2), while the expression of genes for both proteins was hardly changed in various genetic backgrounds tested (Table 2). As far as we know, there have been no such reports. HisA and PurN act on histidine metabolism and purine metabolism, respectively, but their mutations had few or weak effects on genes for histidine synthesis and purine synthesis (Supplementary Materials Tables S5 and S6). Therefore, it is postulated that HisA and PurN are directly or indirectly involved in global transcriptional regulation in *Z. mobilis*.

While our transcriptomic and phenotypic analyses provide compelling evidence for convergent thermal adaptation mechanisms in *Z. mobilis*, we acknowledge certain limitations in our current experimental approach. Functional complementation studies involving the systematic introduction of different mutation combinations into the same strain would provide direct experimental validation of the proposed adaptive mechanisms and potential synergistic effects. Such experiments could determine whether the CHT effects we observed result from independent or interacting mutations. Future work should address whether the mechanistic convergence observed across different mutants, particularly the upregulation of cell wall hydrolase in Z4-80d versus the GroEL/GroES pathway in other strains, reflects fundamental genomic plasticity constraints in *Z. mobilis* that channel adaptation through limited evolutionary trajectories. This limited diversity of thermal resistance mechanisms may reveal important insights into the evolutionary capacity of *Z. mobilis* to adapt to environmental stressors and inform strategies for further strain improvement.

## 4. Materials and Methods

### 4.1. Bacterial Strains and Growth Conditions

The strains used in this study are listed in Table 6. *Z. mobilis* CP4, which was used as a parental strain in this study, was obtained from H. Yanase at Tottori University. *Z. mobilis* cells were grown at 30 °C in 5 mL of YPD medium (0.3% yeast extract, 0.5% peptone, and 3% glucose) under static conditions, and *E. coli* cells were grown at 37 °C in 5 mL of modified Luria–Bertani (LB) medium (1% Bactotryptone, 0.5% yeast extract, 0.5% NaCl, and 80 μL of 5N NaOH) under shaking conditions. If necessary, streptomycin sulfate (80 μg/mL) and rifampicin (3 or 5 μg/mL) were added to the agar plates.

### 4.2. Construction of Single-Mutation Mutants

Each point mutation in the thermoadapted mutants, Z4-80a, Z4-80b, Z4-80c, and Z4-80d, was introduced into CP4 by a procedure similar to that for the construction of mutants of *mutS* or *mutL* reported previously [14]. To prepare the DNA fragment with each point mutation at its center, about a 3000 bp sequence encompassing from the upstream to downstream of each mutation point was amplified by PCR using PrimeSTAR^®^ DNA polymerase (Takara Bio Inc, Kusatsu, Japan), a specific primer set (Supplementary Materials Table S7) and genomic DNA as a template, which was prepared from the cells of each thermoadapted mutant using the boiling method [42]. The resultant PCR fragment was inserted at the *attP* site of the pK18-attP plasmid via a BP reaction (Gateway BP Clonase II enzyme mix, Invitrogen, Waltham, MA, USA) to generate pK18-*attL*-each mutation. The pK18-attP plasmid was constructed by inserting of the *attP* sequence into the *Hin*dIII site of pK18 [14,43]. The recombinant plasmid DNA was then introduced into *E. coli* DH5α to isolate the recombinant plasmid. The plasmid DNA was further introduced into *E. coli* S17-1 [41], and conjugation between *E. coli* S17-1 harboring pK18-*attL*, each mutation and *Z. mobilis* CP4 was carried out as described previously [2]. *E. coli* and *Z. mobilis* cells were cultured until the log phase at 37 °C in LB medium and at 30 °C in YPD medium, respectively, and mixed together at a ratio of 3 to 1. The mixture was washed with LB medium, and cells were suspended in 100 μL of LB medium and incubated at 30 °C under static conditions. After 3 h, 15 μL of the incubated cells was spotted onto YPD plates and incubated at 30 °C. After 4 h, cells were collected, suspended in 400 μL of YPD medium, and spread on YPD plates containing 0.1% (*w*/*v*) acetic acid, 25 µg/mL of kanamycin, 1 mM IPTG (Takara Bio Inc), and 100 µM X-gluc (Bio medical Science, Tokyo, Japan). Blue colonies on the plates were obtained as primary recombinants, and four of the colonies were separately incubated in YPD medium at 30 °C. After 12 h, each incubated sample was spread on YPD plates containing 1 mM IPTG and 100 μM X-gluc. After incubation at 37 °C for 5 d to 7 d, white colonies on the plates were isolated as candidate mutants. Each mutation point was confirmed via direct sequencing with the appropriate primer sets (Supplementary Materials Table S8).

### 4.3. Two-Step Cultivation Assay

To determine the CHT of each strain, we employed a two-phase growth protocol in the YPD medium following our established methodology [14]. Initially, cells were cultured to the late logarithmic phase at temperatures approximating the estimated CHT. Subsequently, an aliquot from this culture was diluted in a fresh medium to achieve an OD_550_ of 0.05 and incubated at the same temperature. When evaluating the impact of specific mutations on thermal resistance, growth experiments were conducted at 38 °C, which is 1 °C above the CHT of the parental strain and 1 °C below the CHTs of the four thermoadapted mutants (as illustrated in Figure 1). Bacterial growth was monitored by measuring optical density at 550 nm. Each CHT determination experiment was performed in triplicate at the respective temperature conditions.

### 4.4. Characterization of Thermoadapted Mutants and Single-Mutation Mutants

To examine the resistance of thermoadapted mutants and single-mutation mutants to polymyxin B, acetic acid, and ethanol, precultured cells that were grown at 30 °C until the mid-log phase were inoculated and cultivated at 30 °C in YPD liquid medium containing polymyxin B, acetic acid, or ethanol at a final concentration of 1 μg/mL, 5% (*w*/*v*) or 10% (*w/v*), respectively. The optical density of the culture at OD_550_ was then measured at 0 h, 12 h, and 24 h. All experiments were performed in triplicate.

### 4.5. Cell Preparation for RNA-Seq

Single colonies of *Z. mobilis* CP4 and mutants were each inoculated into 3 mL of YPD medium and precultured at 30 °C for 12 h under static conditions. Thereafter, the precultured cells were transferred into 30 mL of fresh YPD medium in a 100 mL Erlenmeyer flask at an initial OD_550_ of 0.05 and subsequently cultured at 37 °C for 8 h under static conditions. The cells were collected by centrifugation at 10,000 rpm for 5 min at 4 °C and mixed with 20 mL of chilled phosphate-buffered saline (PBS) by tapping. The mixture was subjected to centrifugation at 10,000 rpm for 5 min at 4 °C and then flushing at 10,000 rpm for 1 min at 4 °C. After all of the supernatant had been removed, cells were kept at −80 °C before being subjected to an RNA preparation process. Three samples were prepared for each strain, and RNA-seq analysis was thus performed in triplicate.

### 4.6. RNA-Seq Analysis

Each cell pellet was placed into a tube containing 0.5 mm glass beads (Tomy Seiko, Inc., Tokyo, Japan) and homogenized in nine volumes of 1-Thioglycerol/Homogenization Solution (Promega, Medison, WS, USA) for 5 min at 3200 rpm using a mixer mill (μT-12, Taitec, Saitama, Japan). After removing the beads, the samples were centrifuged at 12,000 g for 3 min at 4 °C, and the supernatants were immediately used for total RNA extraction. Total RNA was extracted from the supernatants using a Maxwell RSC simplyRNA Tissue Kit (Promega, WS, USA). The concentration of RNA was determined with a Nano Drop ONE (Thermo Fisher Scientific, Inc, Waltham, MA, USA) and its purity was analyzed with TapeStation 4200 High Sensitivity RNA (Agilent Technologies, Inc., Santa Clara, CA, USA) to determine the RINe (RNA Integrity Number equivalent). mRNA from total RNA was purified with the NEBNext rRNA Depletion Kit (Bacteria) (New England Biolabs, NEB, Ipswich, MA, USA). The procedure for complementary DNA (cDNA) libraries was performed with a NEBNext Ultra II RNA Library Prep Kit (NEB) and NEBNext Multiplex Oligos for Illumina (Unique Dual Index), following a previously described method. RNA-seq was performed using the NextSeq 2000 Sequence platform (Illumina, Inc. San Diego, CA, USA). The detailed procedure for RNA-seq has been described previously [44]. All of the data were deposited under accession numbers DRR641198-DRR641245. The reads were trimmed using CLC Genomics Workbench ver. 21.0.3. Trimmed reads were mapped to all the genes in *Z. mobilis* subsp. *mobilis* str. CP4 = NRRL B-14023 (RefSeq accession number: GCA_000498655.1) using CLC Genomics Workbench ver. 21.0.3 (Qiagen, Venlo, Netherlands) with the following parameters: mismatch cost: 2; indel cost: 3; length fraction: 0.8; similarity fraction: 0.8; and the maximum number of hits for a read: 10. The transcripts per million (TPM) value of each gene was estimated as the transcript abundance. The difference in the expression of each gene in thermoadapted or single-mutation mutants was reflected by the ratio of the TPM value in the mutant to that in CP4. For the identification of differentially expressed genes between the WT and mutant cells, the trimmed mean of M-values (TMM) normalization method implemented in the “Generalized linear models (GLM)” algorithm in CLC Genomics Workbench was used. Detailed parameter values used in the algorithm were included in the manufacturer’s product information. Genes with |fold change| > 1.5 and FDR < 0.05 were considered to be highly differentially expressed between WT and mutant cells. Genes with FDR < 0.05 were also considered to be differentially expressed between WT and mutant cells. GO enrichment analysis [45] was performed with ShinyGO 0.80 [46].

## Figures and Tables

**Table 1:** Mutated genes and their mutations in thermoadapted mutants derived from CP4.

Classification	Locus Tag	Old Locus Tag	Gene Product	Z4-80a	Z4-80b	Z4-80c	Z4-80d
Transcriptional regulation	ZCP4_RS00160	ZCP4_0028	Two-component system, sensor histidine kinase for signal transduction		M99I		
	ZCP4_RS02830	ZCP4_0567	RpoB, DNA-directed RNA polymerase subunit beta	R164C		R164C	
Membrane stabilization	ZCP4_RS08695	ZCP4_1739	DgkA, Diacylglycerol kinase	A89V		V223M	L158F
General metabolism	ZCP4_RS02940	ZCP4_0588	PurN: Formyltetrahydrofolate-dependent phosphoribosylglycinamide formyltransferase				L118F
	ZCP4_RS08220	ZCP4_1646	HisA, 1-(5-phosphoribosyl)-5-[(5- phosphoribosylamino)methylideneamino] imidazole-4-carboxamide isomerase		IYDGSL insertion		
Transporter	ZCP4_RS00655	ZCP4_0125	Kup, Potassium transporter	T403A		R597C	
	ZCP4_RS03540	ZCP4_0707	Nitrate/sulfonate/bicarbonate ABC transporter, ATP-binding protein	P438L			
	ZCP4_RS08505	ZCP4_1702	RND family efflux transporter, MFP subunit(HlyD family secretion protein)	E136K		T144M	
	ZCP4_RS08510	ZCP4_1703	Efflux transporter, outer membrane factor lipoprotein, NodT family (TolC family protein)				K462E

**Table 2:** Expression of genes that were mutated in thermoadapted mutants, in various genetic backgrounds.

Locus Tag	Gene Product	Mutant										
		0028M99I	0567R164C	0588L118F	1646aaIns	1739A89V	1739V223M	1739L158F	Z4-80a	Z4-80b	Z4-80c	Z4-80d
		Gene Expression *										
(Z4-80a, Z4-80c, Z4-80d)												
ZCP4_RS08695	DgkA, diacylglycerol kinase family protein	↓↓	↓↓	↓	↓	↑↑	↑↑	-	↑↑	↓	↑↑	↑
(Z4-80a, Z4-80c)												
ZCP4_RS02830	RpoB, DNA-directed RNA polymerase subunit beta	-	↑	-	-	-	-	-	↑	-	↑↑	-
ZCP4_RS00655	Kup, potassium transporter	-	-	-	-	-	-	-	↑	↓	-	-
ZCP4_RS08505	RND family efflux transporter, MFP subunit (HlyD family secretion protein)	↑	↑	-	-	-	-	-	↑	-	↑↑	↑
(Z4-80a)												
ZCP4_RS03540	Nitrate/sulfonate/bicarbonate ABC transporter ATP-binding protein	-	↓	↓	↓	-	-	-	↓↓	-	↓↓	-
(Z4-80b)												
ZCP4_RS00160	HAMP domain-containing sensor histidine kinase (two component system)	-	-	-	-	↑	-	-	↓	↓	-	↓
ZCP4_RS08220	HisA, 1-(5-phosphoribosyl)-5-[(5- phosphoribosylamino)methylideneamino]imidazole-4- carboxamide isomerase	↑	-	-	-	-	-	-	-	-	↑↑	-
(Z4-80d)												
ZCP4_RS02940	PurN, phosphoribosylglycinamide formyltransferase	-	-	-	-	-	-	-	-	-	↓	-
ZCP4_RS08510	TolC family protein (efflux transporter, outer membrane factor lipoprotein)	↑	↑↑	↑	↑	↑	↑	↑	↑↑	-	↑↑	↑

* ↑↑ or ↓↓: FDR < 0.05 and FC > 1.5; ↑ or ↓: FDR < 0.05.

**Table 3:** Expression of *dgkB*, *groEL*, *groES,* and *ZCP4_RS04150* for cell wall hydrolase in various genetic backgrounds.

Locus Tag	Gene Product	Mutant										
		0028M99I	0567R164C	0588L118F	1646aaIns	1739A89V	1739 V223M	1739 L158F	Z4-80a	Z4-80b	Z4-80c	Z4-80d
		Gene Expression *										
ZCP4_RS08515	DgkB: diacylglycerol kinase family lipid kinase	↓↓	↓↓	↓↓	-	-	-	-	↓↓	-	↓↓	↓
ZCP4_RS06260	GroEL: Chaperonin GroEL	-	↑↑	-	↑↑	-	-	↑	↑↑	↑↑	↑	-
ZCP4_RS06265	GroES: Co-chaperone GroES	-	↑↑	-	↑↑	-	-	-	↑↑	↑↑	↑	-
ZCP4_RS04150	Cell wall hydrolase	-	-	↑↑	-	-	-	-	-	-	↑↑	↑↑

* ↑↑ or ↓↓: FDR < 0.05 and FC > 1.5; ↑ or ↓: FDR < 0.05.

**Table 4:** Expression of genes for ABC transporters, efflux pumps, and MFS transporters in various genetic backgrounds.

Locus Tag	Gene Product	Mutant											
		0028M99I	0567R164C	0588L118F	1646aaIns	1739A89V	1739V223M	1739L158F	Z4-80a	Z4-80b	Z4-80c	Z4-80d	Description
		Gene Expression *											
(Operon)													
ZCP4_RS03540	Nitrate/sulfonate/bicarbonate ABC transporter ATP-binding protein	-	↓	↓	↓	-	-	-	↓↓	-	↓↓	-	Mutation in Z4-80a
ZCP4_RS03545	ABC transporter permease subunit	-	↓	↓	↓	↓	-	-	↓↓	↓	↓↓	-	
(Operon)													
ZCP4_RS08495	FUSC family protein	↑	↑	-	-	-	-	-	↑	-	↑↑	↑	
ZCP4_RS08500	DUF1656 domain-containing protein	↑	↑↑	-	-	-	-	-	↑↑	-	↑↑	↑↑	
ZCP4_RS08505	HlyD family secretion protein (efflux transporter, MFP subunit)	↑	↑	-	-	-	-	-	↑	-	↑↑	↑	Mutation in Z4-80a and Z4-80c
ZCP4_RS08510	TolC family protein (efflux transporter, outer membrane factor lipoprotein)	↑	↑↑	↑	↑	↑	↑	↑	↑↑	-	↑↑	↑	Mutation in Z4-80d
(Operon)													
ZCP4_RS02520	HlyD family efflux transporter periplasmic adaptor subunit	↑↑	↑↑	↑	↑↑	-	↑	↑	↑↑	↑↑	↑↑	↑↑	
ZCP4_RS02525	ribosome-associated ATPase/putative transporter RbbA	↑↑	↑	↑	↑	-	↑	-	↑	↑	↑↑	↑	
ZCP4_RS02530	ABC transporter permease	-	↑	-	↑	-	-	-	↑	↑	↑	-	
ZCP4_RS02535	TolC family protein	-	↑	-	↑	-	-	-	↑	↑	↑	-	
(Operon)													
ZCP4_RS07805	ABC transporter permease	↑↑	-	↑↑	-	↑	↑	-	-	-	↑↑	↑↑	
ZCP4_RS07815	ATP-binding cassette domain-containing protein	↑	↑	↑↑	↑	↑	↑	↑	↑	-	↑↑	↑	
													
ZCP4_RS03940	MFS transporter	-	↑	-	-	-	-	-	↑↑	-	↑↑	↑	
ZCP4_RS08380	MFS transporter	↑↑	↑↑	↑↑	↑↑	↑↑	↑↑	-	↑↑	↑↑	↑↑	↑↑	
ZCP4_RS08395	MFS transporter	-	↑↑	↓	-	-	-	-	↑↑	-	↑↑	-	

* ↑↑ or ↓↓: FDR < 0.05 and FC > 1.5; ↑ or ↓: FDR < 0.05.

**Table 5:** Shared DEGs between two mutants, of which mutations affected the expression of relatively many genes.

Upregulated DEGs										
Mutant and a pair for comparison	0567R164C	0028M99I	1646aaIns	0588L118F	0567R164C and 0028M99I	0567R164C and 1646aaIns	0567R164C and 0588L118F	0028M99I and 1646aaIns	0028M99I and 0588L118F	1646aaIns and 0588L118F
Number of genes	165	48	48	84	37	33	48	16	28	23
Shared genes * (%)					79%	69%	57%	33%	60%	48%
**Downregulated DEGs**										
Mutant and a pair for comparison	0567R164C	0028M99I	1646aaIns	0588L118F	0567R164C and 0028M99I	0567R164C and 1646aaIns	0567R164C and 0588L118F	0028M99I and 1646aaIns	0028M99I and 0588L118F	1646aaIns and 0588L118F
Number of genes	299	215	87	260	162	69	178	60	155	70
Shared genes * (%)					75%	79%	68%	69%	72%	80%

* The percentage was calculated using the smaller number of DEGs of the two mutants as the denominator.

**Table 6:** Bacterial strains used in this study.

Strain	Description	Reference
*Zymomonas mobilis*		
CP4		H. Yanase [14]
Z4-80a	Thermoadapted mutant of CP4	[14]
Z4-80b	Thermoadapted mutant of CP4	[14]
Z4-80c	Thermoadapted mutant of CP4	[14]
Z4-80d	Thermoadapted mutant of CP4	[14]
0567R164C	CP4 with a missense mutation (R to C at position 164 in the product of ZCP4_RS02830 (old locus tag ZCP4_0567)) from Z4-80a	This study
1739A89V	CP4 with a missense mutation (A to V at position 89 in the product of ZCP4_RS08695 (old locus tag ZCP4_1739)) from Z4-80a	This study
0125T403A	CP4 with a missense mutation (T to A at position 403 in the product of ZCP4_RS00655 (old locus tag ZCP4_0125)) from Z4-80a	This study
0707P438L	CP4 with a missense mutation (P to L at position 438 in the product of ZCP4_RS03540 (old locus tag ZCP4_0707)) from Z4-80a	This study
1702E136K	CP4 with a missense mutation (E to K at position 136 in the product of ZCP4_RS08505 (old locus tag ZCP4_1702)) from Z4-80a	This study
0028M99I	CP4 with a missense mutation (M to I at position 99 in the product of ZCP4_RS00160 (old locus tag ZCP4_0028)) from Z4-80b	This study
1646aaIns	CP4 with an insertion mutation (IYDGSL at position 230 in the product of ZCP4_RS08220 (old locus tag ZCP4_1646)) from Z4-80b	This study
1739V223M	CP4 with a missense mutation (V to M at position 223 in the product of ZCP4_RS08695 (old locus tag ZCP4_1739)) from Z4-80c	This study
0125R597C	CP4 with a missense mutation (R to C at position 597 in the product of ZCP4_RS00655 (old locus tag ZCP4_0125)) from Z4-80c	This study
1702T144M	CP4 with a missense mutation (T to M at position 144 in the product of ZCP4_RS08505 (old locus tag ZCP4_1702)) from Z4-80c	This study
1739L158F	CP4 with a missense mutation (L to F at position 158 in the product of ZCP4_RS08695 (old locus tag ZCP4_1739)) from Z4-80d	This study
0588L118F	CP4 with a missense mutation (L to F at position 118 in the product of ZCP4_RS02940 (old locus tag ZCP4_0588)) from Z4-80d	This study
1703K462E	CP4 with a missense mutation (K to E at position 462 in the product of ZCP4_RS08510 (old locus tag ZCP4_1703)) from Z4-80d	This study
*Escherichia coli*		
DH5*λ*	F^−^, ϕ80*lacZ*ΔM15,Δ(*lacZYA-argF*) U169, *deoR*, *recA*1, *endA*1, *hsdR*17(r_K_^−^, m_K_^+^), *phoA*, *supE*44, *λ^−^*, *thi*-1, *gyrA*96, *relA*1	Takara Bio
S17-1	F^−^, *recA*, *pro*, *thi*, *hsdR*, [RP4-2 Tc::Mu Km::Tn*7* Tra^+^ Tp^r^ Sm^r^]	[41]

## Data Availability

All relevant data are within the paper and its Supporting Information Files. The RNA-seq data underlying the study are available on the NCBI GenBank repository within BioProject number PRJDB2030.

## Supplementary Materials

The supporting information can be downloaded at: https://www.mdpi.com/article/10.3390/ijms26073052/s1.

## Abbreviations

The following abbreviations are used in this manuscript:

RNA-seq	RNA sequencing
CHT	Critical high temperature
DEGs	Differentially expressed genes
GO	Gene Ontology
LPS	Lipopolysaccharide
IMP	Inner membrane protein
PAP	Periplasmic adapter protein
OMF	Outer membrane factor
MFS	Major facilitator superfamily
ROS	Reactive oxygen species
LB	Luria–Bertani
cDNA	Complementary DNA
TPM	Transcripts per million
TMM	Trimmed mean of M-values
GLM	Generalized linear models
