# Supplementary material for: Limited Diversity of Thermal Adaptation to a Critical Temperature in Zymomonas mobilis: Evidence from Multiple-Parallel Laboratory Evolution Experiments
Source: Int J Mol Sci. 2025 Mar 26;26(7):3052. doi: 10.3390/ijms26073052 (PMC11989028; doi:10.3390/ijms26073052)
Supplement: Supplementary file 1 [file ijms-26-03052-s001.zip › ijms-3492743-supplementary.pdf]

Supplementary Materials

Low diversity of thermal adaptation to a critical temperature in *Zymomonas mobilis* suggested by multiple-parallel laboratory evolution experiments

Sornsiri Pattanakittivorakul[1], Shun Kato[1], Takashi Kuga[1], Tomoyuki Kosaka[1, 2], Minenosuke Matsutani[3], Masayuki Murata[1], Morio Ishikawa[4], Kankanok Charoenpunthuwong[5], Pornthap Thanonkeo[5,6], and Mamoru Yamada[1, 2,*]

[1] Graduate School of Sciences and Technology for Innovation, Yamaguchi University, Yamaguchi 753-8515, Japan; sornkiri@gmail.com (S.P.); sh.kato@ikedatohka.co.jp (S.K.); t.kuga0724@gmail.com (T.K.); murama0054@gmail.com (M.M.)
[2] Research Center for Thermotolerant Microbial Resources, Yamaguchi University, Yamaguchi 753-8515, Japan; tkosaka@yamaguchi-u.ac.jp (T.K.)
[3] NODAI Genome Research Center, Tokyo University of Agriculture, Tokyo 156-8502, Japan; mine@yamaguchi-u.ac.jp (M.M.)
[4] Department of Bioscience, Tokyo University of Agriculture, Tokyo 156-8502, Japan; m1ishika@nodai.ac.jp (M.I.)
[5] Department of Biotechnology, Faculty of Technology, Khon Kaen University, Thailand; cchomkan@gmail.com (K.C.)
[6] Fermentation Research Center for Value Added Agricultural Products, Khon Kaen University, Thailand; portha@kku.ac.th (P.T.)
*Correspondence: m-yamada@yamaguchi-u.ac.jp; Tel.: +81-83-933-5869

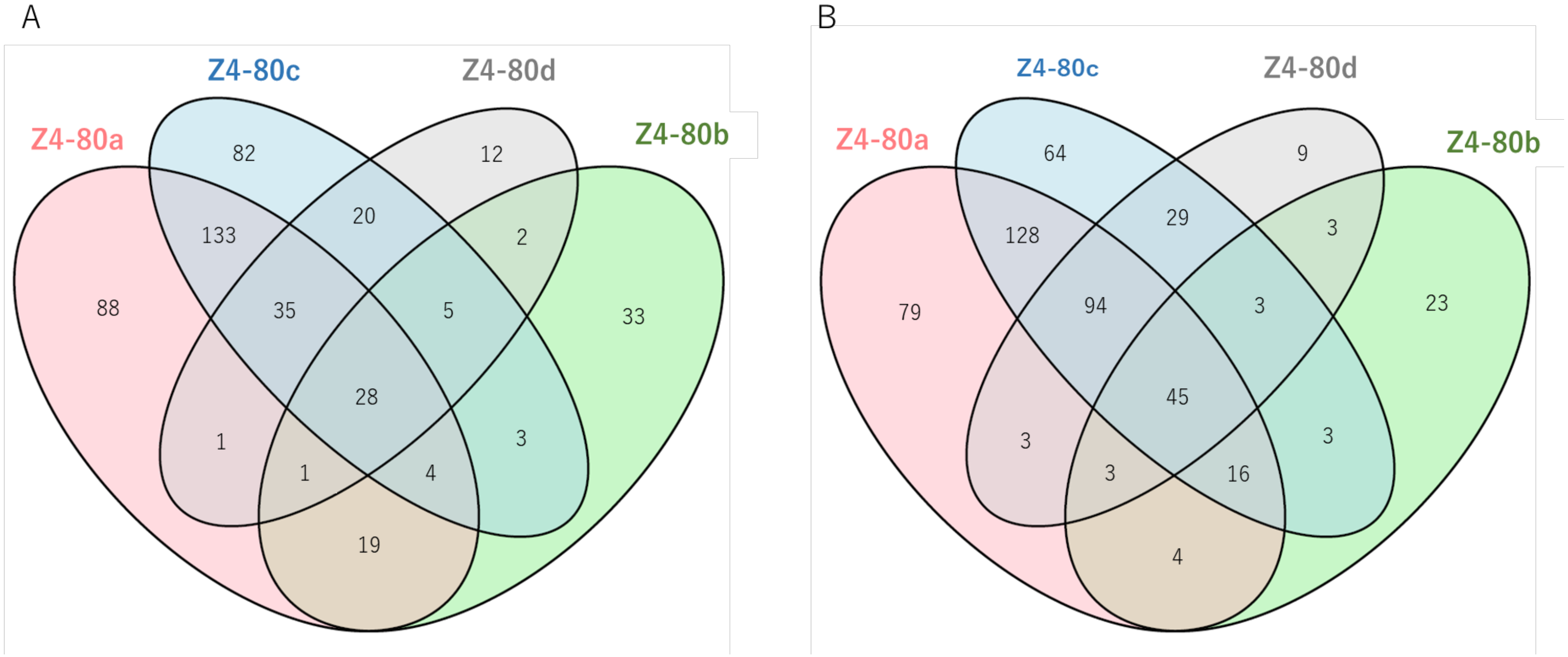

Figure S1. Comparison of DEGs of Z4-80a, Z4-80b, Z4-80c, and Z4-80d. Venn diagram analysis was performed for upregulated DEGs (A) and downregulated DEGs (B) of Z4-80a, Z4-80b, Z4-80c, and Z4-80d. There were 72 DEGs conserved in 4 thermoadapted mutants, including 28 upregulated and 45 downregulated genes which are shown in the center of each diagram.

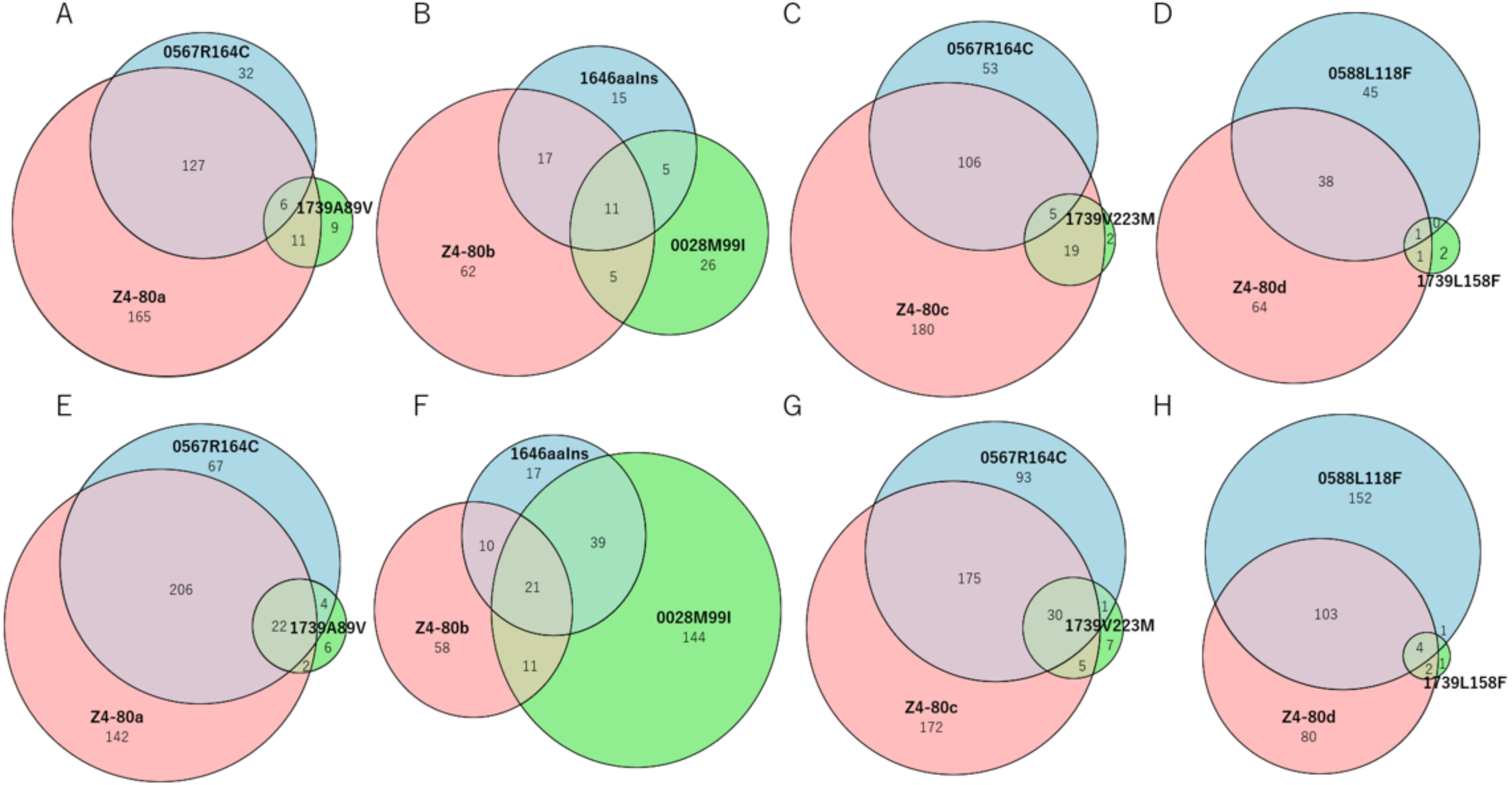

Figure S2. Comparison DEGs of two mutants mainly contributing to thermal resistance and the parental thermoadapted mutant. Venn diagram analysis was performed for upregulated DEGs (upper row) and downregulated DEGs (lower row) of Z4-80a, 0567R164C and 1739A89V (A, E); Z4-80b, 0028M99I and 1646aaIns (B, F); Z4-80c, 0567R164C and 1739V223M (C, G); and Z4-80d, 0588 and 1739L158F (C, H).

Table S1. Upregulated DEGs and downregulated DEGs shared by Z4-80a, Z4-80b, Z4-80c and Z4-80d.

Upregulated DEGs ($log_2 > 1$)

Locus tag	Gene product
ZCP4_RS00565	50S ribosomal protein L9
ZCP4_RS00570	30S ribosomal protein S18
ZCP4_RS00575	30S ribosomal protein S6
ZCP4_RS01355	N-acetyltransferase
ZCP4_RS01535	translation initiation factor IF-1
ZCP4_RS02520	HlyD family efflux transporter periplasmic adaptor subunit
ZCP4_RS03725	50S ribosomal protein L15
ZCP4_RS04660	DUF882 domain-containing protein
ZCP4_RS05145	ferredoxin family protein
ZCP4_RS05540	phosphatase PAP2 family protein
ZCP4_RS05790	hemolysin family protein
ZCP4_RS05975	(Fe-S)-binding protein
ZCP4_RS05980	lactate utilization protein B
ZCP4_RS05985	LUD domain-containing protein
ZCP4_RS06160	hypothetical protein
ZCP4_RS06235	hypothetical protein
ZCP4_RS09720	50S ribosomal protein L32
ZCP4_RS07245	FKBP-type peptidyl-prolyl cis-trans isomerase
ZCP4_RS07345	YhjD/YihY/BrkB family envelope integrity protein
ZCP4_RS07370	ATP phosphoribosyltransferase regulatory subunit
ZCP4_RS07905	SDR family oxidoreductase
ZCP4_RS07970	tRNA-Lys
ZCP4_RS09735	50S ribosomal protein L35
ZCP4_RS08150	50S ribosomal protein L20
ZCP4_RS08375	hypothetical protein
ZCP4_RS08380	MFS transporter
ZCP4_RS08640	M23 family metallopeptidase
ZCP4_RS08795	30S ribosomal protein S4

Downregulated genes ($log_2 < -1$)

Locus_tag	Gene product
ZCP4_RS09690	entericidin A/B family lipoprotein
ZCP4_RS00900	hypothetical protein
ZCP4_RS01220	septal ring lytic transglycosylase RlpA family protein
ZCP4_RS02160	chemotaxis-specific protein-glutamate methyltransferase CheB
ZCP4_RS02440	UDP-glucose/GDP-mannose dehydrogenase family protein
ZCP4_RS02450	hypothetical protein
ZCP4_RS02690	tetratricopeptide repeat protein
ZCP4_RS03140	YnbE family lipoprotein
ZCP4_RS03295	flagellin
ZCP4_RS03595	pyridoxal phosphate-dependent aminotransferase
ZCP4_RS04210	glycine zipper 2TM domain-containing protein
ZCP4_RS04345	ATP-dependent Clp protease ATP-binding subunit ClpA
ZCP4_RS05360	phosphomethylpyrimidine synthase ThiC
ZCP4_RS05505	tetratricopeptide repeat protein
ZCP4_RS05550	S1/P1 nuclease
ZCP4_RS05820	carbohydrate porin
ZCP4_RS05935	YcgN family cysteine cluster protein
ZCP4_RS06185	3-methyl-2-oxobutanoate hydroxymethyltransferase
ZCP4_RS06295	primosomal protein N'
ZCP4_RS06475	alkene reductase
ZCP4_RS06635	flavodoxin FldA
ZCP4_RS06640	DUF2023 family protein
ZCP4_RS06660	ABC transporter ATP-binding protein
ZCP4_RS07310	GTP cyclohydrolase II
ZCP4_RS07320	zinc-binding alcohol dehydrogenase family protein
ZCP4_RS07500	tRNA lysidine(34) synthetase TilS
ZCP4_RS07505	tetratricopeptide repeat protein
ZCP4_RS07630	succinyl-diaminopimelate desuccinylase
ZCP4_RS07635	TonB-dependent siderophore receptor
ZCP4_RS07640	hypothetical protein
ZCP4_RS08060	ferrous iron transport protein B
ZCP4_RS08065	FeoA family protein

ZCP4_RS08320	electron transfer flavoprotein subunit beta/FixA family protein
ZCP4_RS08325	FAD-binding protein
ZCP4_RS08605	(2Fe-2S)-binding protein
ZCP4_RS09000	phage tail assembly protein
ZCP4_RS09005	phage major tail tube protein
ZCP4_RS09235	TonB-dependent receptor
ZCP4_RS09260	TonB-dependent receptor
ZCP4_RS09265	type II toxin-antitoxin system YafQ family toxin
ZCP4_RS09270	type II toxin-antitoxin system RelB/DinJ family antitoxin
ZCP4_RS09365	ABC transporter ATP-binding protein
ZCP4_RS09370	ABC transporter ATP-binding protein
ZCP4_RS09570	DUF2312 domain-containing protein
ZCP4_RS09575	hypothetical protein

Table S2. GO enrichment (Top 20) in upregulated and downregulated DEGs in Z4-80a, Z4-80b, Z4-80c, and Z4-80d.

Z4-80a upregulated DEGs

Enrichment FDR	Number of genes	Pathway Genes	Fold Enrichment	Pathways
4.1E-33	133	1652	2.4	Cellular anatomical entity
2.1E-21	113	1508	2.2	Cellular process
7.1E-17	100	1355	2.2	Metabolic process
2.8E-16	93	1213	2.3	Catalytic activity
3.1E-13	85	1151	2.2	Cellular metabolic process
8.6E-11	81	1167	2.1	Organic substance metabolic process
1.9E-10	76	1070	2.1	Binding
4.9E-09	73	1072	2	Intracellular
1.9E-10	69	910	2.3	Cytoplasm
1.8E-07	67	1014	2	Primary metabolic process
1.8E-04	58	1006	1.7	Nitrogen compound metabolic process
3.1E-13	54	511	3.2	Membrane
3.2E-06	52	739	2.1	Ion binding
1.4E-05	52	783	2	Organic cyclic compound binding
1.4E-05	52	783	2	Heterocyclic compound binding
1.3E-04	48	757	1.9	Biosynthetic process
1.1E-03	44	734	1.8	Organic substance biosynthetic process
1.0E-08	43	453	2.8	Cell periphery
1.3E-03	43	719	1.8	Cellular biosynthetic process
2.3E-03	43	739	1.7	Organonitrogen compound metabolic process

Z4-80b upregulated DEGs

Enrichment FDR	Number of Genes	Pathway Genes	Fold Enrichment	Pathways
2.7E-12	54	1652	2.4	Cellular anatomical entity
2.2E-03	36	1508	1.8	Cellular process
1.4E-03	32	1213	1.9	Catalytic activity
8.0E-03	32	1355	1.7	Metabolic process
4.1E-04	31	1070	2.1	Binding
2.3E-04	29	910	2.3	Cytoplasm
2.1E-03	29	1072	2	Intracellular
3.4E-02	26	1151	1.7	Cellular metabolic process
3.9E-04	25	739	2.5	Ion binding
8.9E-05	22	511	3.2	Membrane
1.0E-02	22	783	2.1	Organic cyclic compound binding
1.0E-02	22	783	2.1	Heterocyclic compound binding
1.1E-03	18	453	2.9	Cell periphery
7.2E-04	17	392	3.2	Plasma membrane
6.8E-03	16	450	2.6	Nucleotide binding
6.8E-03	16	450	2.6	Nucleoside phosphate binding
1.6E-02	16	507	2.3	Small molecule binding
1.3E-02	15	442	2.5	Anion binding
1.2E-02	13	346	2.8	Integral component of membrane
1.8E-03	12	227	3.9	Oxidation-reduction process

Z4-80c upregulated DEGs

Enrichment FDR	Number of Genes	Pathway Genes	Fold Enrichment	Pathways
1.5E-35	135	1652	2.5	Cellular anatomical entity
3.9E-19	109	1508	2.2	Cellular process
6.4E-13	92	1355	2	Metabolic process
7.3E-14	88	1213	2.2	Catalytic activity
3.3E-09	77	1167	2	Organic substance metabolic process
5.7E-07	71	1151	1.9	Cellular metabolic process
5.4E-21	65	511	3.8	Membrane
7.7E-07	65	1014	1.9	Primary metabolic process
6.0E-06	65	1070	1.8	Binding
1.4E-04	61	1072	1.7	Intracellular
7.6E-06	58	910	1.9	Cytoplasm
4.2E-17	56	453	3.7	Cell periphery
2.4E-03	54	1006	1.6	Nitrogen compound metabolic process
4.2E-17	49	346	4.3	Integral component of membrane
3.2E-14	48	392	3.7	Plasma membrane
2.3E-03	44	757	1.8	Biosynthetic process
4.3E-03	42	734	1.7	Organic substance biosynthetic process
4.6E-03	42	739	1.7	Ion binding
2.1E-02	41	783	1.6	Organic cyclic compound binding
2.1E-02	41	783	1.6	Heterocyclic compound binding

Z4-80d upregulated DEGs

Enrichment FDR	Number of Genes	Pathway Genes	Fold Enrichment	Pathways
1.6E-07	46	1652	2.1	Cellular anatomical entity
3.2E-03	33	1355	1.9	Metabolic process
2.3E-02	33	1508	1.7	Cellular process
1.6E-02	29	1213	1.8	Catalytic activity
4.8E-02	24	1070	1.7	Binding
2.7E-02	23	910	1.9	Cytoplasm
8.9E-08	17	189	6.9	Mixed, incl. membrane, and periplasmic space
3.7E-02	15	511	2.3	Membrane
2.7E-02	10	227	3.4	Oxidation-reduction process
3.1E-02	9	208	3.3	Oxidoreductase activity
1.8E-03	8	88	7	Mixed, incl. mfs transporter superfamily, and dna-binding transcription factor a
2.7E-02	8	154	4	Cellular amino acid biosynthetic process, and nucleoside monophosphate metabolic
1.1E-05	7	28	19.2	Mixed, incl. major facilitator superfamily, and lactate metabolic process
2.7E-02	6	84	5.5	Iron-sulfur cluster binding
2.7E-02	6	85	5.4	Metal cluster binding
4.2E-02	6	106	4.4	Mixed, incl. membrane, and cellular ketone metabolic process
4.2E-02	6	107	4.3	Mixed, incl. siderophore transport, and polysaccharide biosynthetic process
2.7E-02	5	52	7.4	Mixed, incl. siderophore transport, and glycosyl hydrolase, five-bladed beta-pro
2.7E-02	5	59	6.5	Mixed, incl. siderophore transport, and alpha/beta hydrolase fold
1.7E-03	4	12	25.6	Mixed, incl. lactate metabolic process, and sapc

Z4-80a downregulated DEGs

Enrichment FDR	Number of Genes	Pathway Genes	Fold Enrichment	Pathways
3.9E-29	143	1652	2.2	Cellular anatomical entity
1.1E-10	105	1508	1.8	Cellular process
2.3E-06	86	1355	1.6	Metabolic process
7.6E-06	78	1213	1.7	Catalytic activity
2.3E-06	73	1070	1.8	Binding
8.6E-03	64	1167	1.4	Organic substance metabolic process
1.5E-15	63	511	3.2	Membrane
5.1E-16	60	453	3.4	Cell periphery
1.2E-02	59	1072	1.4	Intracellular
8.1E-04	57	910	1.6	Cytoplasm
3.8E-04	50	739	1.7	Ion binding
4.4E-03	48	783	1.6	Organic cyclic compound binding
4.4E-03	48	783	1.6	Heterocyclic compound binding
2.5E-10	43	349	3.2	Intrinsic component of membrane
1.7E-06	38	392	2.5	Plasma membrane
3.1E-11	37	245	3.9	Transport
5.4E-11	37	250	3.8	Establishment of localization
4.1E-10	37	272	3.5	Localization
3.3E-05	35	395	2.3	Hydrolase activity
3.9E-03	35	507	1.8	Small molecule binding

Z4-80b downregulated DEGs

Enrichment FDR	Number of Genes	Pathway Genes	Fold Enrichment	Pathways
6.0E-06	41	1652	2	Cellular anatomical entity
3.4E-03	32	1508	1.7	Cellular process
6.5E-05	20	511	3.2	Membrane
4.6E-04	17	453	3	Cell periphery
2.2E-06	16	245	5.3	Transport
2.6E-06	16	250	5.2	Establishment of localization
6.8E-06	16	272	4.7	Localization
1.1E-04	16	349	3.7	Intrinsic component of membrane
3.4E-04	15	346	3.5	Integral component of membrane
1.3E-05	13	183	5.7	Transmembrane transporter activity
1.6E-05	13	188	5.6	Transporter activity
6.6E-05	12	183	5.3	Transmembrane transport
1.1E-07	11	72	12.3	Mixed, incl. transmembrane transport, and hopanoid metabolic process
1.3E-05	11	123	7.2	Ion transport
1.4E-03	11	227	3.9	Oxidation-reduction process
4.5E-02	11	392	2.3	Plasma membrane
2.4E-03	10	208	3.9	Oxidoreductase activity
1.1E-09	9	22	33	Mixed, incl. efflux transmembrane transporter activity, and rnd efflux pump, mem
1.4E-08	9	30	24.2	Mixed, incl. efflux transmembrane transporter activity, and barrel-sandwich doma
7.2E-04	8	107	6	Mixed, incl. siderophore transport, and polysaccharide biosynthetic process

Z4-80c downregulated DEGs

Enrichment FDR	Number of Genes	Pathway Genes	Fold Enrichment	Pathways
6.2E-35	156	1652	2.3	Cellular anatomical entity
7.2E-09	105	1508	1.7	Cellular process
3.2E-08	96	1355	1.7	Metabolic process
3.1E-07	86	1213	1.7	Catalytic activity
2.6E-05	73	1072	1.7	Intracellular
4.8E-05	72	1070	1.6	Binding
2.8E-07	71	910	1.9	Cytoplasm
4.2E-02	63	1167	1.3	Organic substance metabolic process
3.3E-12	60	511	2.9	Membrane
6.1E-12	55	453	3	Cell periphery
7.9E-03	47	739	1.6	Ion binding
1.8E-10	45	349	3.1	Intrinsic component of membrane
4.6E-02	45	783	1.4	Organic cyclic compound binding
4.6E-02	45	783	1.4	Heterocyclic compound binding
8.9E-10	38	272	3.4	Localization
1.9E-10	37	245	3.7	Transport
3.2E-10	37	250	3.6	Establishment of localization
1.1E-04	35	392	2.2	Plasma membrane
3.4E-07	33	273	2.9	Biological regulation
6.3E-04	33	395	2	Hydrolase activity

Z4-80d downregulated DEGs

Enrichment FDR	Number of Genes	Pathway Genes	Fold Enrichment	Pathways
5.6E-14	66	1652	2.3	Cellular anatomical entity
1.0E-02	41	1508	1.6	Cellular process
3.0E-02	36	1355	1.5	Metabolic process
6.9E-03	33	1070	1.8	Binding
3.4E-02	33	1213	1.6	Catalytic activity
1.2E-02	32	1072	1.7	Intracellular
5.8E-03	30	910	1.9	Cytoplasm
4.4E-03	21	511	2.4	Membrane
6.8E-03	19	453	2.4	Cell periphery
3.0E-03	15	273	3.2	Biological regulation
1.4E-02	15	346	2.5	Integral component of membrane
2.2E-03	14	227	3.6	Oxidation-reduction process
6.9E-03	11	181	3.5	Reg. of cellular process
6.9E-03	11	182	3.5	Mixed, incl. membrane, and periplasmic space
8.7E-03	11	190	3.4	Response to stimulus
1.4E-02	11	208	3.1	Oxidoreductase activity
3.6E-02	11	245	2.6	Transport
4.2E-02	11	250	2.6	Establishment of localization
2.2E-03	10	107	5.4	Mixed, incl. siderophore transport, and polysaccharide biosynthetic process
4.4E-02	9	183	2.9	Transmembrane transport

Table S3. Upregulated and downregulated DEGs shared by Z4-80a and 1739A89V; Z4-80c and 1739V223M; and Z4-80d and 1739L158F.

A pair of mutants	Shared DEGs	Locus tag	Gene product
ZM80a and 1739A89V	Upregulated	ZCP4_RS00370	aminotransferase class I/II-fold pyridoxal phosphate-dependent enzyme
		ZCP4_RS00375	acyl carrier protein
		ZCP4_RS00975	thioredoxin-disulfide reductase
		ZCP4_RS06910	glycosyltransferase family 39 protein
		ZCP4_RS07140	hypothetical protein
		ZCP4_RS07765	ribonuclease HI
		ZCP4_RS07770	homoserine kinase
		ZCP4_RS08660	sterol desaturase family protein
		ZCP4_RS08690	DUF2141 domain-containing protein
		ZCP4_RS08695	acylglycerol kinase family protein
		ZCP4_RS08700	hypothetical protein
	Downregulated	ZCP4_RS04965	response regulator transcription factor
		ZCP4_RS08605	(2Fe-2S)-binding protein
ZM80c and 1739V223M	Upregulated	ZCP4_RS00320	LacI family DNA-binding transcriptional regulator
		ZCP4_RS00375	acyl carrier protein
		ZCP4_RS00975	thioredoxin-disulfide reductase
		ZCP4_RS01915	hypothetical protein
		ZCP4_RS01920	glycosyl hydrolase 108 family protein
		ZCP4_RS01925	hypothetical protein
		ZCP4_RS01930	hypothetical protein
		ZCP4_RS10010	hypothetical protein
		ZCP4_RS04455	PBSX family phage terminase large subunit
		ZCP4_RS04480	glycoside hydrolase family 68 protein
		ZCP4_RS04485	glycoside hydrolase family 68 protein
		ZCP4_RS05980	lactate utilization protein B
		ZCP4_RS05985	LUD domain-containing protein
		ZCP4_RS06910	glycosyltransferase family 39 protein
		ZCP4_RS07140	hypothetical protein

		ZCP4_RS07770	homoserine kinase
		ZCP4_RS08690	DUF2141 domain-containing protein
		ZCP4_RS08695	acylglycerol kinase family protein
		ZCP4_RS08700	hypothetical protein
	Downregulated	ZCP4_RS02450	hypothetical protein
		ZCP4_RS02745	RNA polymerase sigma factor RpoH
		ZCP4_RS04265	SUF system Fe-S cluster assembly regulator
		ZCP4_RS04965	response regulator transcription factor
		ZCP4_RS08605	(2Fe-2S)-binding protein
ZM80d and 1739L158F	Upregulated	ZCP4_RS04485	glycoside hydrolase family 68 protein
	Downregulated	ZCP4_RS07065	FAD-binding oxidoreductase
		ZCP4_RS07150	peroxiredoxin

Table S4. Comparison of upregulated DEGs shared by Z4-80d and 0588L118F with upregulated DEGs shared by Z4-80a and 0567R164C; Z4-80b and 0028M99I or 1646aaIns; and Z4-80c and 0567R164C.

Locus tag	Gene product	Z4-80a and 0567R164C	Z4-80b and 0028M99I or 1646aaIns	Z4-80c and 0567R164C
ZCP4_RS00025	OmpA family protein	○*	-	○
ZCP4_RS00385	glutamine-hydrolyzing GMP synthase	○	-	○
ZCP4_RS00565	50S ribosomal protein L9	○	○	○
ZCP4_RS00570	30S ribosomal protein S18	○	○	○
ZCP4_RS00575	30S ribosomal protein S6	○	○	○
ZCP4_RS00690	transcriptional regulator NrdR	-	-	-
ZCP4_RS01475	anaerobic ribonucleoside-triphosphate reductase activating protein	-	-	-
ZCP4_RS02315	alanine--tRNA ligase	○	-	○
ZCP4_RS02670	hypothetical protein	-	-	-
ZCP4_RS03705	30S ribosomal protein S11	○	-	○
ZCP4_RS03710	30S ribosomal protein S13	○	-	○
ZCP4_RS03815	50S ribosomal protein L4	-	-	-
ZCP4_RS03820	50S ribosomal protein L3	-	-	-
ZCP4_RS03825	30S ribosomal protein S10	○	-	○
ZCP4_RS04125	formate--tetrahydrofolate ligase	-	○	-
ZCP4_RS04150	cell wall hydrolase	-	-	-
ZCP4_RS04660	DUF882 domain-containing protein	○	○	○
ZCP4_RS05000	response regulator	○	-	○
ZCP4_RS05020	HU family DNA-binding protein	○	-	○
ZCP4_RS05180	50S ribosomal protein L27	○	-	○
ZCP4_RS05300	hypothetical protein	○	-	○
ZCP4_RS05790	hemolysin family protein	○	○	○
ZCP4_RS05990	heparinase II/III family protein	-	-	○
ZCP4_RS05995	ribulose-phosphate 3-epimerase	-	-	-
ZCP4_RS06440	hypothetical protein	○	-	○
ZCP4_RS06905	dihydroxy-acid dehydratase	○	-	-
ZCP4_RS06960	M1 family metallopeptidase	○	○	-

ZCP4_RS07165	hypothetical protein	○	-	○
ZCP4_RS07420	DUF721 domain-containing protein	-	○	○
ZCP4_RS07805	ABC transporter permease	-	-	-
ZCP4_RS07920	DUF2474 domain-containing protein	○	-	○
ZCP4_RS07925	cytochrome d ubiquinol oxidase subunit II	○	-	○
ZCP4_RS07930	cytochrome ubiquinol oxidase subunit I	○	-	○
ZCP4_RS09735	50S ribosomal protein L35	○	○	○
ZCP4_RS08150	50S ribosomal protein L20	○	○	○
ZCP4_RS08375	hypothetical protein	○	○	○
ZCP4_RS08380	MFS transporter	○	○	○
ZCP4_RS08595	Fur family transcriptional regulator	-	-	-
ZCP4_RS08640	M23 family metallopeptidase	○	○	○

*○: upregulated; -: not upregulated.

Table S5. Expression of genes for purine metabolism in various genetic backgrounds.

				Mutant										
				0028M99I	0567R164C	0588L118F	1646aaIns	1739 A89V	1739 V223M	1739L158F	Z4-80a	Z4-80b	Z4-80c	Z4-80d
Locus tag	Gene	EC No	Gene product	Gene expression*										
ZCP4_RS02940	*purN*	2.1.2.2	phosphoribosylglycinamide formyltransferase										↓	
ZCP4_RS05955	*purH*	2.1.2.3	bifunctional phosphoribosylaminoimidazolecarboxamide formyltransferase/IMP cyclohydrolase	↑	↑	↑	↑				↑↑	↑	↑↑	↑
ZCP4_RS02935	*purM*	6.3.3.1	phosphoribosylformylglycinamidine cyclo-ligase										↓	
ZCP4_RS03155	*purB*	4.3.2.2	adenylosuccinate lyase	↑	↑↑	↑	↑				↑↑		↑↑	↑
ZCP4_RS04795	*purD*	6.3.4.13	phosphoribosylamine--glycine ligase	↑	↑↑	↑	↑				↑↑	↑	↑↑	↑
ZCP4_RS07990	*purF*	2.4.2.14	amidophosphoribosyltransferase		↑						↑↑		↑↑	
ZCP4_RS08085	*purQ*	6.3.5.3	phosphoribosylformylglycinamidine synthase subunit PurQ								↑		↑	
ZCP4_RS08090	*purS*		phosphoribosylformylglycinamidine synthase subunit PurS		↓	↓		↓	↓		↑		↑	
ZCP4_RS08400			purine nucleoside permease		↑						↑↑		↑↑	↑
ZCP4_RS08550	*purE*	5.4.99.18	5-(carboxyamino)imidazole ribonucleotide mutase	↑	↑		↑				↑↑	↑	↑↑	↑
ZCP4_RS01740			purine nucleoside permease	↑	↑								↑↑	
ZCP4_RS02435	*purL*		phosphoribosylformylglycinamidine synthase subunit PurL	↑	↑↑	↑					↑↑		↑↑	↑
ZCP4_RS01375		6.3.2.6	phosphoribosylaminoimidazolesuccinocarboxamide synthase		↑		↑				↑		↑	↑

*↑↑ or ↓↓: FDR < 0.05 and FC > 1.5; ↑ or ↓: FDR < 0.05. Up and down arrows represent up-regulation and down-regulation, respectively.

Table S6. Expression of genes for histidine metabolism in various genetic backgrounds.

Locus tag	Gene	EC No	Gene product	Mutant										
				0028M99I	0567R164C	0588L118F	1646aaIns	1739 A89V	1739 V223M	1739L158F	Z4-80a	Z4-80b	Z4-80c	Z4-80d
				Gene expression*										
ZCP4_RS08020	*hisG*	2.4.2.17	ATP phosphoribosyltransferase	↓					↓				↓	
ZCP4_RS08230		3.6.1.31	phosphoribosyl-ATP diphosphatase		↑						↑↑		↑	
ZCP4_RS00810	*hisI*	3.5.4.19	phosphoribosyl-AMP cyclohydrolase	↓	↓	↓								
ZCP4_RS08220	*hisA*	5.3.1.16	1-(5-phosphoribosyl)-5-[(5-phosphoribosylamino)methylideneamino]imidazole-4-carboxamide isomerase	↑									↑↑	
ZCP4_RS08215	*hisH*	4.3.2.10	imidazole glycerol-phosphate synthase	↑	↑	↑					↑↑		↑↑	↑
ZCP4_RS08210	*hisB*	4.2.1.19	imidazoleglycerol-phosphate dehydratase subunit HisB	↑									↑↑	
ZCP4_RS08140		2.7.6.1	ribose-phosphate pyrophosphokinase, PRPP synthetase	↑↑	↑↑	↑	↑				↑↑		↑↑	↑↑
		3.1.3.15	histidinol-phosphatase											
ZCP4_RS08225	*hisF*	4.2.1.19	imidazoleglycerol-phosphate dehydratase subunit HisF	↑	↑						↑		↑↑	
ZCP4_RS04270	*hisC*	2.6.1.9	histidinol-phosphate transaminase	↑	↑				↑		↑↑	↑	↑	
ZCP4_RS08015	*hisD*	1.1.1.23	histidinol dehydrogenase								↓		↓	

*↑↑ or ↓↓: FDR < 0.05 and FC > 1.5; ↑ or ↓: FDR < 0.05. Up and down arrows represent up-regulation and down-regulation, respectively.

Table S7. Primers used for BP reaction in construction of single-mutation mutants.

Primer	Sequence (5’------>3’)
ZCP4_0028-attB F	GGGGACAAGTTTGTACAAAAAAGCAGGCTCGCGTGTCAGGTTGGTACGGAG
ZCP4_0028-attB R	GGGGACCACTTTGTACAAGAAAGCTGGGTCCTATCAGGCGCAAAGCAAGG
ZCP4_1739-attB F	GGGGACAAGTTTGTACAAAAAAGCAGGCTCGATAACTTATCCCGACCGCCC
ZCP4_1739-attB R	GGGGACCACTTTGTACAAGAAAGCTGGGTCGTGCCTTTGGCAAGTAAGCG
ZCP4_0588-attB F	GGGGACAAGTTTGTACAAAAAAGCAGGCTCGTCCGCTTCACATGCAACCTT
ZCP4_0588-attB R	GGGGACCACTTTGTACAAGAAAGCTGGGTCATGCCTTTTGATCGGCAGGT
ZCP4_0567-attB F	GGGGACAAGTTTGTACAAAAAAGCAGGCTCGCTCCTCGACATTGCTGGTGT
ZCP4_0567-attB R	GGGGACCACTTTGTACAAGAAAGCTGGGTCAGTGATCTGTTCGCGAGGTG
ZCP4_0125-attB F	GGGGACAAGTTTGTACAAAAAAGCAGGCTCGGGCTTGGTCAGCCTCATCTT
ZCP4_0125-attB R	GGGGACCACTTTGTACAAGAAAGCTGGGTCCATCGACCCGATGGGCAATA
ZCP4_0707-attB F	GGGGACAAGTTTGTACAAAAAAGCAGGCTCGTGGCATTGTTGTCATGGCCT
ZCP4_0707-attB R	GGGGACCACTTTGTACAAGAAAGCTGGGTCCGCTTGGCCTATGTCGGTAT
ZCP4_1702-attB F	GGGGACAAGTTTGTACAAAAAAGCAGGCTCGGACGATGTTCCCTGTGCTGTT
ZCP4_1702-attB R	GGGGACCACTTTGTACAAGAAAGCTGGGTCCAGGCTTTCACCATCACCAAC
ZCP4_1703-attB F	GGGGACAAGTTTGTACAAAAAAGCAGGCTCGTGCCGCAACGTATTCCTGTT
ZCP4_1703-attB R	GGGGACCACTTTGTACAAGAAAGCTGGGTCATACCCATATTGTCGCCGCT
ZCP4_1646-attB F	GGGGACAAGTTTGTACAAAAAAGCAGGCTCGGGAAGCAAGGCCGAATACCT
ZCP4_1646-attB R	GGGGACCACTTTGTACAAGAAAGCTGGGTCCGCGAATAATGCGGGCAAA

Table S8. Primers used for confirmation of single-mutation mutants.

Primer	Sequence (5’----->3’)
ZCP4_0028_F	GCCATTTCATTCTGGCTACG
ZCP4_0028_R	ATACCGCCTATCAGGATGATG
ZCP4_1739_F	AAGAAGAACAGCGAGGGTAC
ZCP4_1739_R	TGCGGATCTTGTTGAAACAC
ZCP4_1739_F	CGACAGCCATATCGTCAAAC
ZCP4_1739_R	TCAGGCCATCAAGATCAGTC
ZCP4_0588_F	AGGATTATATGGCGACCAAGG
ZCP4_0588_R	GATTCTTTCACCTTCGCTCG
ZCP4_0567_F	CAGCTTTCAGCGACATAAGG
ZCP4_0567_R	TGAGGACGGATATGTTTCAGG
ZCP4_0125_F	GTGTGCCGTTGATTATTCTCG
ZCP4_0125_R	GGGATAAGTGTCTGACGAGAG
ZCP4_0125_F	ATGGCTTCTATCGCCTGATC
ZCP4_0125_R	CGGAGATTGGTCTCTAGTGG
ZCP4_0707_F	ACATCGGTTTGGCTTGTTAC
ZCP4_0707_R	TTACCTCAGCCCAGAAATCG
ZCP4_1702_F	GATGTTTCCGGTTTGGTCAC
ZCP4_1702_R	CCCATGTAAAGGTTGGGTTG
ZCP4_1703_F	AGCCGCTCTTACAACCTATAC
ZCP4_1703_R	CATTCCTGCGTGATCTTGTTC
ZCP4_1646_F	TTCTGTCACTGAGATGGCTG
ZCP4_1646_R	CGGAATTAACGGCGACTTTG
